# The Impact of Quarantine on Physical Activity, Body Weight, and Eating Behaviors During the COVID-19 Pandemic in Saudi Arabia

**DOI:** 10.7759/cureus.56460

**Published:** 2024-03-19

**Authors:** Raghad M Alsaqqa, Raghad M Alasmari, Rawan A Altalhi, Nuha Filfilan

**Affiliations:** 1 College of Medicine, Taif University, Taif, SAU; 2 Public Health and Preventive Medicine, Taif University, Taif, SAU

**Keywords:** eating behaviors, saudi arabia, pandemic, covid-19, body weight, physical activity, quarantine

## Abstract

Background

Since February 2020, the number of coronavirus disease 2019 (COVID-19) cases in Saudi Arabia has been rising. The Saudi Arabian government implemented strict lockdown measures in March 2020 in an effort to stop the spread of the virus. Globally, changes in socioeconomic levels and health during quarantine have been reported. Additional detrimental effects of the stay-at-home mandate include less physical activity among adult populations, anxiety, stress, and emotional eating. This study aimed to assess the impact of quarantine during the COVID‑19 pandemic on physical activity practice and weight.

Methodology

This cross-sectional study was conducted using a newly developed validated online questionnaire survey. The data were collected during quarantine in June 2020, including each adult who lived in Saudi Arabia during the quarantine. The data were reviewed and analyzed using SPSS version 21 (IBM Corp., Armonk, NY, USA).

Results

A total of 4,970 eligible participants completed the study survey. About 78.1% of the study participants performed physical activity inside the house during the pandemic. Overall, 30.9% reported increased body weight during the lockdown, and the most reported cause of increased body weight was feeling bored and empty. Regarding factors associated with physical activity and weight changes, 82.2% of participants aged 25-30 years performed physical activity during the pandemic. Increased weight was significantly higher among participants aged more than 30 years.

Conclusions

This study shows an increased level of physical activity among the study participants during the COVID-19 pandemic, especially among younger, female, and unmarried participants. More than half of the participants reported weight change, either weight gain or weight loss. Weight gain was higher in older participants. Regarding eating habits, there was an increased tendency toward eating a healthy diet.

## Introduction

At the beginning of 2020, the world was facing the coronavirus disease 2019 (COVID-19) pandemic, which began in the city of Wuhan in China with an unknown cause of pneumonia cases [1-3], later discovered to be caused by severe acute respiratory syndrome coronavirus 2. However, it quickly spread and became a pandemic in March 2020 [4,5]. The World Health Organization has become concerned with alarming levels of spread, severity, and level of infection [6]. The pandemic impacted the world and caused significant damage, both socially and economically [7]. At the beginning of the pandemic, no vaccine or treatment had been created, and quarantine became the best option and recommendation to stop the rapid spread of infection. Governments around the world have adopted strategies to limit the spread of the virus, and they have enforced restrictions on outdoor activities [8]. The Saudi government has imposed strict measures to limit the spread of the virus, including the use of protective measures such as wearing face masks and gloves, travel restrictions, closure of schools and universities, and curfew [9]. This situation has led to a change in the lifestyle and habits of the Saudi population, including factors that impact body weight such as physical inactivity, psychological situation, nutritional habits, and sleep behavior.

Physical activity (PA) plays a significant role in physical and mental health besides well-being, and it contributes to systemic inflammation, obesity, and chronic disease prevention. Up to five million deaths a year can be prevented if the worldwide population becomes more active. In 2018, the World Health Assembly agreed on an international goal to reduce physical inactivity by 15% by 2030 in alignment with the Sustainable Development Goals [10]. In the context of the COVID-19 pandemic, regular PA enhances immunity and protects against viral infections by decreasing the risk, duration, or severity of viral infection [11]. Furthermore, unclear routines and loss of time and space boundaries, for example, mealtime, and separation between work and home space increase the risk of developing an eating disorder [12]. In addition, obesity has been associated with a worse prognosis of viral infection, higher mortality rates, more severe clinical course, and hospitalization [13]. In the case of coronavirus, body mass index (BMI) was significantly high in patients with complications and severe forms of infection [14].

Globally, the negative impact of quarantine on PA and consumption of unhealthy food has been proven [15,16]. According to Fitbit data during the COVID-19 pandemic, there was a dramatic decline in PA globally [17]. A cross-sectional study showed significantly lower levels of PA among the Sicilian active population during the COVID-19 quarantine in comparison to before the quarantine, especially among the male and overweight group [3]. Another study proved the remarkable reduction of PA levels among southern Croatia adolescents, especially boys. The PA level of boys may have contributed to less participation in formal sports and organized recreational activities [18].

Moreover, reduced PA along with an unhealthy diet contributed to weight gain during quarantine [19]. The experience of Poland shows that a remarkable percentage of people changed their eating habits by increasing their eating and intake of snacks, as well as a change in weight [20]. In an Italian study, 46.1% of the sample had changed their eating habits negatively by increasing their intake of comfort foods, which increased the weight of 19.5% of the sample. On the positive side, high consumption of vegetables and fruits was observed in 21.2% of the sample [21]. Furthermore, during the COVID-19 pandemic lockdown in Brazil, respondents who engaged in sedentary behavior were more likely to consume an unhealthy diet [22].

A previous study showed that 22% of the study sample had gained 5-10 pounds. This increase in weight was related to many factors, including decreased PA, lack of sleep, snacking after dinner, and eating due to stress or the smell of food. On the other hand, 15% of the sample lost 5-10 pounds [23]. In another study, Rundle et al. expected that the COVID-19 pandemic may amplify obesity risk factors in children due to school closings [24]. Pietrobelli et al. tested this hypothesis and found unsatisfactory changes in the lifestyle and habits of children and adolescents associated with the risk of weight gain and obesity, thereby proving the findings of Rundle et al. [25].

Regarding Saudi Arabia, in a cross-sectional study done during the COVID-19 quarantine, about 45% of participants reported eating more snacks, and 28% had gained weight [26]. In another study about the impact of COVID-19 home quarantine on lifestyle, the prevalence of participants who used to walk daily significantly decreased, and the prevalence of participants who often consumed snacks between meals increased, while the prevalence of participants who never ate fruits and vegetables significantly increased during home quarantine [27]. Furthermore, a cross-sectional study conducted in Jeddah City, Saudi Arabia, found that major changes in eating behavior were reported in people exposed to the full COVID-19 quarantine period rather than the partial curfew [28].

Despite the intense influence of the COVID-19 pandemic on people’s lifestyles worldwide, data are scarce on the impact of quarantine on the Saudi population’s lifestyle during the pandemic. Therefore, this study aimed to measure the impact of quarantine on body weight, eating behaviors, and PA among the Saudi population during the COVID-19 pandemic as well as the factors affecting it.

## Materials and methods

Study design and procedure

This descriptive, cross-sectional study was conducted using an online questionnaire survey performed via the Typeform web survey platform. A research proposal and a request letter were submitted to the Dean of the College of Medicine and the Vice Dean of Scientific Research for approval before the study was conducted. Ethical approval was obtained from the Research Ethical Committee at Taif University (approval number: 42-0033).

The primary objectives were to estimate the general rate of increase or decrease in weight during quarantine and to compare the rate of change in weight in both sexes at different ages. In addition, we aimed to assess whether quarantine resulted in increased food consumption, snacking, and cooking. The study also estimated the changes in the level of PA during quarantine.

Study participants

The participants in the study completed the online questionnaire between June 2020 and June 2021. The number of participants was 4,970 at a confidence interval of 95% and a margin of error of 2%. The study included individuals of both sexes, aged 15 years old and above, who were in Saudi Arabia during the COVID-19 quarantine, irrespective of whether they were citizens or residents. We excluded those who were under 15 years old, pregnant, and who were outside Saudi Arabia during the COVID-19 quarantine.

Questionnaire

The newly developed, self-administered, English-language version of the questionnaire to assess PA and eating habits consisted of three sections. Section A consisted of personal and disease details. Section B comprised details of working (job) status and PAs. Section C included eating habits and their effect on health during the pandemic. The drafted English-language version was then subjected to validation. A standardized methodology was followed in the validation of the questionnaire, which included focus group discussion, expert evaluation, pilot study, reliability, and validity assessment. The content validity, face validity, and construct validity of the developed questionnaire were examined.

Data analysis

The data were collected, reviewed, and then entered into SPSS version 21 (IBM Corp., Armonk, NY, USA). All statistical methods used were two-tailed with an alpha level of 0.05, considering significance if the p-value was less than or equal to 0.05. Descriptive analysis was done by prescribing frequency distribution and percentages for study variables, including participants’ personal data, work data, medical history, and habits with their BMI. Moreover, participants’ PA practice before and during the pandemic, along with their reported types of activity, were tabulated. Sleep patterns, dietary habits, and weight changes during the COVID-19 pandemic were also tabulated, while reasons for weight changes were graphed. Cross-tabulation was used to identify factors associated with practicing PA and weight changes during the COVID-19 lockdown using the Pearson chi-square test and the exact probability test for small frequency distributions.

## Results

A total of 4,970 eligible participants completed the study questionnaire. Participants ages ranged from 12 to 80 years, with a mean age of 26.9 ± 8.9 years. Overall, 86.3% were female, and 93.3% were Saudi. About 38% were from Makkah Province, 25.3% from Riyadh Province, 11.2% from Eastern Province, and 10.3% from Qassim Province. Regarding educational level, 76% were university graduates. Overall, 33.9% of the participants were married. Concerning BMI, 49.9% had a normal body weight, 23.9% were overweight, and 18.6% were obese. In addition, 8.2% had a chronic disease, mainly hypertension (49.8%), diabetes mellitus (27.8%), and 31.3% had other diseases (Table 1).

**Table 1:** Sociodemographic characteristics of the study participants.

Personal data	No	%
Age in years		
<20	756	15.2%
20–24	1,863	37.5%
25–30	1,046	21.0%
>30	1,305	26.3%
Gender		
Female	4,287	86.3%
Male	683	13.7%
Nationality		
Saudi	4,637	93.3%
Non-Saudi	333	6.7%
Place of residence		
Makkah Province	1,890	38.0%
Riyadh Province	1,259	25.3%
Eastern Province	557	11.2%
Qassim Province	512	10.3%
Al Madinah Province	361	7.3%
Aseer Province	103	2.1%
Tabuk Province	101	2.0%
Ha'il Province	39	0.8%
Northern Borders Province	39	0.8%
Al Jawf Province	38	0.8%
Jazan Province	36	0.7%
Al Baha Province	28	0.6%
Najran Province	7	0.1%
Educational level		
Uneducated	5	0.1%
Intermediate or secondary school	836	16.8%
University graduate	3,779	76.0%
Postgraduate	350	7.0%
Marital status		
Married	1,684	33.9%
Unmarried	3,286	66.1%
Body mass index		
Underweight	380	7.6%
Normal weight	2,478	49.9%
Overweight	1,190	23.9%
Obese	922	18.6%
Do you suffer from any chronic diseases?		
Yes	407	8.2%
No	4,563	91.8%
If yes, mention the disease		
Diabetes mellites	113	27.8%
Hypertension	202	49.8%
Others	127	31.3%
Chronic liver disease	111	27.3%
Chronic heart disease	10	2.5%
Cancer	6	1.5%

Table 2 presents work and PA among participants before the COVID‑19 pandemic. Overall, 24.6% of the study participants were employed, 25.4% worked during the lockdown, and 24.2% worked remotely. In addition, 42.6% stated that their work required them to make a movement effort, and 21.7% reported a need for strong effort at their work. During lockdown, 82.2% spent more than 20 hours at home. Overall, 73.4% reported having outdoor space in their house (yard, roof, garden). Considering PA, 67.8% performed PA inside the house before the pandemic. The most reported sports done before the pandemic included walking (79.8%), resistance exercises (30.4%), and dancing (27.7%). Furthermore, 44.9% of those who did any activity did it four to five times per week, and 35.7% did it daily. Overall, 50.1% performed the activity inside the home, and 49.9% performed it outside the home.

**Table 2:** Work and physical activity among the study participants before the COVID‑19 pandemic.

Work and activity before COVID-19	No	%
Job status		
Employed	1,222	24.6%
Non-employed	3,748	75.4%
Did you work during the lockdown?		
Yes	310	25.4%
No	490	40.1%
Sometimes	126	10.3%
Work remotely	296	24.2%
Does your work require you to make a movement effort?		
Yes	521	42.6%
No	701	57.4%
How much motor effort do you expend at work?		
Weak	31	6.0%
Average	377	72.4%
Strong	113	21.7%
Do you own outdoor space in your house (yard, roof, garden)?		
Yes	3,646	73.4%
No	1,324	26.6%
Did you do any physical activity inside the house before the pandemic?		
Yes	3,372	67.8%
No	1,598	32.2%
Did you do any kind of sport before the pandemic?		
No	1,213	24.4%
Rarely	846	17.0%
Sometimes	2,052	41.3%
Regularly	859	17.3%
What kind of sport did you do before the pandemic?		
Walking	2,995	79.8%
Resistance exercises	1,141	30.4%
Dancing	1,039	27.7%
Swimming	330	8.8%
Bike riding	309	8.2%
Playing football	173	4.6%
Others	80	2.1%
How many times per week?		
Once	8	0.9%
2–3 times	158	18.4%
4–5 times	386	44.9%
Daily	307	35.7%
Where did you play sports before the pandemic?		
Inside the house	1,881	50.1%
Outside the house	1,876	49.9%

Table 3 shows the PA levels among participants during the COVID‑19 pandemic. Overall, 78.1% of the study participants performed physical activity inside the house during the pandemic. A total of 42.3% practiced sports four to five times a week and 46.1% did daily. The most practiced sports during the pandemic included walking (74%), resistance exercises (45.2%), and dancing (29.6%). The vast majority of the participants (86.8%) played sports inside the home during the quarantine.

**Table 3:** Physical activity among participants during the COVID‑19 pandemic.

Physical activity during COVID-19	No	%
Do you do any physical activity inside the house during the pandemic?		
Yes	3,884	78.1%
No	1,086	21.9%
Did you do any kind of sport during the pandemic?		
No	1,327	26.7%
Rarely	948	19.1%
Sometimes	1,826	36.7%
Regularly	869	17.5%
How many times per week?		
Once	6	0.7%
2–3 times	94	10.8%
4–5 times	368	42.3%
Daily	401	46.1%
What kind of sport did you do during the pandemic?		
Walking	2,690	74.0%
Resistance exercises	1,644	45.2%
Dancing	1,075	29.6%
Bike riding	280	7.7%
Swimming	156	4.3%
Others	89	2.4%
Playing football	76	2.1%
Where did you play sports during the pandemic?		
Inside the house	3,163	86.8%
Outside the house	480	13.2%

Table 4 presents sleep habits and weight changes during the COVID‑19 pandemic. The vast majority of the study participants spent more than 20 hours at home during the lockdown. Regarding sleep hours during the lockdown, 50.8% reported sleeping for seven to nine hours, and 19.9% reported sleeping for more than nine hours. Overall, 65.5% of the participants experienced eating habit changes during the lockdown, mainly preparing baked goods (pastries) and sweets at home (50.2%), reducing fast food intake (46.7%), increasing daily food intake (40%), reducing daily food intake (36.7%), increasing the consumption of vegetables and fruits (35.8%), increasing the consumption of frozen and canned foods (22.1%), and using nutritional supplements and vitamins (13.8%). Moreover, 30.9% reported increased body weight during the lockdown, 30.5% experienced decreased body weight, and 38.5% had no weight change. For those who experienced increased weight, it was 1-2 kg among 34.1%, 3-4 kg among 34.5%, and 5-6 kg among 12.6%. For those who lost weight, it was 1-2 kg among 34.3% of them, 3-4 kg among 35.8%, and 5-6 kg among 11.5%.

**Table 4:** Sleep habits and weight changes during the COVID‑19 pandemic.

Sleep habits and weight changes	No	%
How many hours do you spend at home during the lockdown period?		
8–12 hours	227	4.6%
12–16 hours	228	4.6%
16–20 hours	429	8.6%
>20 hours	4,086	82.2%
Your sleep hours during the day during the lockdown period		
<4 hours	140	2.8%
5–6 hours	1,317	26.5%
7–9 hours	2,525	50.8%
>9 hours	988	19.9%
Did your eating habits change during the lockdown?		
Yes	3,256	65.5%
No	1,714	34.5%
How did your eating habits change?		
Preparing baked goods (pastries) and sweets at home	1,635	50.2%
Reducing fast food intake	1,519	46.7%
Increasing daily food intake	1,303	40.0%
Reducing daily food intake	1,194	36.7%
Increasing the consumption of vegetables and fruits	1,165	35.8%
Increasing the consumption of frozen and canned foods	721	22.1%
Using nutritional supplements and vitamins	449	13.8%
Reducing the consumption of frozen and canned foods	365	11.2%
Eating more fast food	247	7.6%
Reducing the consumption of vegetables and fruits	151	4.6%
Others	22	0.7%
Did you notice any change in weight during the pandemic?		
Increased	1,538	30.9%
Decreased	1,518	30.5%
No changes	1,914	38.5%
The amount of increase in kilograms		
1–2	525	34.1%
3–4	531	34.5%
5–6	194	12.6%
>6	105	6.8%
I do not know	183	11.9%
The amount of decrease in kilograms		
1–2	520	34.3%
3–4	544	35.8%
5–6	175	11.5%
>6	150	9.9%
I do not know	129	8.5%

**Figure 1:**
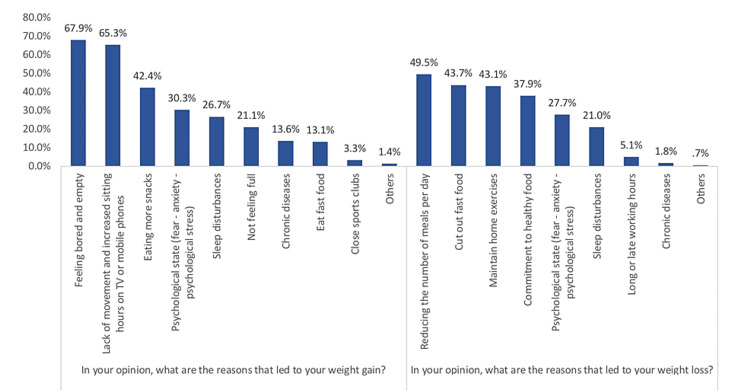
Reasons for weight changes among the study participants during the COVID‑19 pandemic.

Figure 1 illustrates the reasons for weight changes among the study participants during the COVID‑19 pandemic. Regarding the reasons for increased weight, most reported feeling bored and empty (67.9%), lack of movement, and increased sitting hours on TV or mobile phones (65.3%). About the reasons for weight loss, the most reported reasons were reducing the number of meals per day (49.5%) and cutting out fast food (43.7%).

Table 5 presents the factors associated with practicing PA during the COVID‑19 lockdown. Overall, 82.2% of participants aged 25-30 years performed PA during the pandemic versus 73.1% of those less than 20 years old, with a recorded statistical significance (p = 0.001). Moreover, 81% of females did PA versus 60.3% of males (p = 0.001). PA during the pandemic was practiced by 87.1% of non-Saudi participants compared to 77.5% of Saudi participants (p = 0.001). Likewise, 85.7% of married people performed activities during the pandemic versus 74.3% of unmarried people (p = 0.001). Additionally, 78% of those who did not work during the lockdown practiced activity compared to 62.9% of those who were working (p = 0.001). Around 79.5% of overweight and 78.1% of obese participants did PA during the quarantine. Overall, 95.3% of participants who performed PA inside the house before the pandemic still did it during the pandemic versus 41.9% who did not (p = 0.001).

**Table 5:** Factors associated with practicing physical activity during the COVID‑19 lockdown. P: Pearson chi-square test; ^: exact probability; * p < 0.05 (significant).

Factors	Physical activity during the lockdown				P-value
	Yes		No		
	No	%	No	%	
Age in years					0.001*
<20	553	73.1%	203	26.9%	
20–24	1,411	75.7%	452	24.3%	
25–30	860	82.2%	186	17.8%	
>30	1,060	81.2%	245	18.8%	
Gender					0.001*
Female	3,472	81.0%	815	19.0%	
Male	412	60.3%	271	39.7%	
Nationality					0.001*
Saudi	3,594	77.5%	1,043	22.5%	
Non-Saudi	290	87.1%	43	12.9%	
Educational level					0.078^
Uneducated	5	100.0%	0	0.0%	
Intermediate or secondary school	659	78.8%	177	21.2%	
University graduate	2,975	78.7%	804	21.3%	
Postgraduate	245	70.0%	105	30.0%	
Marital status					0.001*
Married	1,443	85.7%	241	14.3%	
Unmarried	2,441	74.3%	845	25.7%	
Job status					0.064
Employed	892	73.0%	330	27.0%	
Non-employed	2,992	79.8%	756	20.2%	
Body mass index					0.592
Underweight	292	76.8%	88	23.2%	
Normal weight	1,926	77.7%	552	22.3%	
Overweight	946	79.5%	244	20.5%	
Obese	720	78.1%	202	21.9%	
Did you work during the lockdown?					0.001*
Yes	195	62.9%	115	37.1%	
No	382	78.0%	108	22.0%	
Sometimes	94	74.6%	32	25.4%	
Work remotely	221	74.7%	75	25.3%	
Does your work require you to make a movement effort?					0.056
Yes	395	75.8%	126	24.2%	
No	497	70.9%	204	29.1%	
Did you do any physical activity inside the house before the pandemic?					0.001*
Yes	3,214	95.3%	158	4.7%	
No	670	41.9%	928	58.1%	

Table 6 presents the factors associated with weight change during the COVID‑19 lockdown. Increased weight was significantly higher among participants aged more than 30 years (36.8%), married participants (37.5%), obese participants (43.4%), those who worked remotely (36.1%), those who did not practice PA before the pandemic (31.7%), and those who did not practice PA during the pandemic (40%).

**Table 6:** Factors associated with weight change during the COVID‑19 lockdown. P: Pearson chi-square test; ^: exact probability; * p < 0.05 (significant).

Factors	Did you notice any change in weight during the pandemic period?						P-value
	Increased		Decreased		No changes		
	No	%	No	%	No	%	
Age in years							0.001*
<20	200	26.5%	281	37.2%	275	36.4%	
20–24	541	29.0%	619	33.2%	703	37.7%	
25–30	317	30.3%	309	29.5%	420	40.2%	
>30	480	36.8%	309	23.7%	516	39.5%	
Gender							0.691
Female	1,335	31.1%	1,310	30.6%	1,642	38.3%	
Male	203	29.7%	208	30.5%	272	39.8%	
Nationality							0.069
Saudi	1,419	30.6%	1,434	30.9%	1,784	38.5%	
Non-Saudi	119	35.7%	84	25.2%	130	39.0%	
Educational level							0.078^
Uneducated	2	40.0%	0	0.0%	3	60.0%	
Intermediate or secondary school	250	29.9%	263	31.5%	323	38.6%	
University graduate	1,155	30.6%	1,167	30.9%	1,457	38.6%	
Postgraduate	131	37.4%	88	25.1%	131	37.4%	
Marital status							0.001*
Married	632	37.5%	370	22.0%	682	40.5%	
Unmarried	906	27.6%	1,148	34.9%	1,232	37.5%	
Job status							0.085
Employed	408	33.4%	352	28.8%	462	37.8%	
Non-employed	1,130	30.1%	1,166	31.1%	1,452	38.7%	
Body mass index							0.001*
Underweight	55	14.5%	126	33.2%	199	52.4%	
Normal weight	664	26.8%	793	32.0%	1,021	41.2%	
Overweight	419	35.2%	388	32.6%	383	32.2%	
Obese	400	43.4%	211	22.9%	311	33.7%	
Did you work during the lockdown period?							0.013*
Yes	89	28.7%	75	24.2%	146	47.1%	
No	171	34.9%	152	31.0%	167	34.1%	
Sometimes	41	32.5%	38	30.2%	47	37.3%	
Worked remotely	107	36.1%	87	29.4%	102	34.5%	
Does your work require you to make a movement effort?							0.932
Yes	177	34.0%	149	28.6%	195	37.4%	
No	231	33.0%	203	29.0%	267	38.1%	
Did you do any physical activity inside the house before the pandemic?							0.003*
Yes	1,032	30.6%	990	29.4%	1,350	40.0%	
No	506	31.7%	528	33.0%	564	35.3%	
Did you do any kind of sport during the pandemic?							0.001*
No	531	40.0%	248	18.7%	548	41.3%	
Sometimes	554	30.3%	564	30.9%	708	38.8%	
Rarely	349	36.8%	205	21.6%	394	41.6%	
Regularly	104	12.0%	501	57.7%	264	30.4%	

## Discussion

In this study, most participants were young and unmarried, with nearly normal body weight for half of the participants. Moreover, the vast majority (75.4%) were not employed. Of the employed participants, 42.6% said that their work needed effort, mainly at an average level. Regarding the home environment, 73.4% of the study participants owned outdoor space in their houses. All these factors (young age, unemployment, lack of diseases, and having home space) motivated them to practice PA, which was reported by about two-thirds of the participants, with mainly walking and resistance exercises. This was done nearly daily by 35.7% of the participants, and four to five times a week by 44.9%, which implied high PA performance before the pandemic. This was consistent with what was reported by the general authority of statistics in Saudi Arabia, which showed that 48.2% of the Kingdom’s population practices PA for at least 30 minutes per week. The results also showed a significant difference between the percentages of males and females who practiced PA for at least 30 minutes per week, as the percentage of males was 54.8%, and the percentage of females was only 38.3% [29]. Another systematic review conducted by Al-Hazzaa [30] assessed that the prevalence of physical inactivity varied based on the population being assessed, region, age, gender, kind of PA instrument used, and physical inactivity criteria, ranging from 26% to 85% for Saudi males and from 43% to 91% for Saudi females.

During the COVID‑19 pandemic, this study revealed that about 78% of the participants performed PA inside the house, which was regular among about 17% of the participants. Walking and resistance exercises were the most practiced activities, with around 40% of participants performing PA four to five times per week or daily. Due to the quarantine, the vast majority practiced sports inside their houses, and this was due to the presence of spaces in houses (garden or roof). This implies that the rate of performed PA increased during the quarantine, which was in contrast to the findings of Rasheed et al. [31], where more than half of the participants reported less PA than before the COVID-19 pandemic. Similarly, Park et al. [32] conducted a review study including studies in different world regions and demonstrated a substantial correlation between COVID-19 and increases in sedentary behavior and decreases in walking, mobility, and PA. A few studies also revealed conflicting findings, such as an increase in the use of parks and trails and a rise in recreational activities among particular demographic groups. In Saudi Arabia, Hamed et al. [33] conducted a study with no statistically significant difference between pre- and post-COVID-19 respondents in terms of PA. The estimated higher PA among the current study participants may be due to more female participants who had the time, and most of them were unemployed, which means their lifestyle was nearly unaffected by maintaining their traditional PA patterns.

Regarding eating habits and weight change, about 65.5% of the participants experienced eating habit changes during the lockdown. The nature of changed habits was contradictory, as increased preparation of baked goods (pastries) and sweets at home, reduced fast food intake, reduced daily food intake, and increased consumption of vegetables and fruits were reported. On the other hand, an increase in daily food intake and an increase in the consumption of frozen and canned foods were also reported. Weight change was reported by 60% of the participants, where nearly half of them (30.9%) experienced increased body weight, and the other half experienced decreased body weight (30.5%). The weight change was mostly around 1-4 kg. Similar results were reported by Bakhsh et al. [26], who found that about 28% of the participants gained weight as a result of their increased eating, as indicated by 40% and 45% of individuals, respectively. Regarding eating habits in this study, only 7% of the participants said they ate meals from restaurants compared to the majority who said they ate home-cooked (73%) and healthy meals (47%). The two primary motivators for food behavior changes were time constraints (40%) and feelings of emptiness and boredom (44%). Other studies have reported weight gain of nearly 3-4.5 kg during the quarantine [20]. Weight gain is anticipated because of the overall drop in PA during a quarantine, which is linked to restricting people’s ability to go to work, the gym, parks, and even perform their regular daily activities. Additionally, the psychological distress brought on by spending months confined to one’s house, as well as the worry about novelty and the rapid development of COVID-19 [34]. Furthermore, higher food intake was linked to the experienced worldwide lockdown, according to earlier reports from Poland, Italy, and the United Kingdom [20,21,35]. The reported decreased weight in our study participants was mainly due to increased PA besides avoiding fast food intake. More engagement on social media with a lack of movement decreased the number of daily meals, which was reported as the main cause by the study respondents.

Although this study has an adequate sample size from various regions that represent the Saudi population, it has some limitations. As a result of COVID-19 and lockdown restrictions, the only way to collect data was through an online survey, which had some disadvantages such as recall bias, misunderstanding of survey questions, and the anthropometric measurements being measured incorrectly. In addition, as the survey was distributed online through social media, people without Internet access could not answer the questionnaire. A phone call or physical interview would be more accurate. Furthermore, other variables were missed in this study, such as psychological and socioeconomic level changes, that may influence the lifestyle of participants. We recommend considering them in future studies.

## Conclusions

This study revealed an increased level of PA among the study participants during the COVID-19 pandemic, mostly in younger, female, unmarried participants who had outdoor space in their houses. Regarding weight, more than half of the participants reported weight changes of mostly around 1-4 kg during the quarantine; gaining weight was higher in participants aged above 30 years. Furthermore, changes in feeding habits, such as increased intake of homemade food, having supplements and vitamins, and eating a diet higher in fruits and vegetables and lower in other unhealthy foods, were also reported.

## Appendices

English version of the questionnaire to assess physical activity and eating habits during quarantine.

1. Do you agree with the answer to this questionnaire, given that all information will remain confidential and used only by researchers in this research?

Yes (move to Question No. 2)

No (moves to the end of the questionnaire)

2. Gender:

Male

Female

3. Age: (open answer)

4. Weight in kilograms: (open answer)

5. Height in centimeters: (open answer)

6. Nationality:

Saudi

Non-Saudi

7. Place of residence:

Riyadh Province

Makkah Province

Al-Madinah Province

Qassim Province

Eastern Province

Aseer Province

Tabuk Province

Ha'il Province

Northern Borders Province

Jazan Province

Najran Province

Al-Baha Province

Al Jawf Province

Others: (mention it)

8. Educational level:

Uneducated

Intermediate or secondary school

University

Postgraduate

9. Marital status:

Married

Unmarried

10. Do you suffer from any chronic diseases?

No (move to Question No. 12)

Yes (move to Question No. 11)

11. What is it?

Diabetes

Hypertension

Chronic heart disease

Chronic liver disease

Asthma or chronic lung diseases

Cancer

Other (mention it)

12. Job status:

Employee (move to Question No. 13)

Not employed (move to Question No. 16)

13. Did you work during the curfew period?

Yes

No

Sometimes

Work remotely

14. Does your work's nature require you to make a movement effort (such as a policeman, a nurse, a teacher, a coach…)?

Yes (move to Question No. 15)

No (move to Question No. 16)

15. How much motor effort do you expend at work?

Weak

Average

strong

16. How many hours do you spend at home during the curfew period?

8-12 hours

12-16 hours

16-20 hours

The whole day

17. Do you own outdoor space in your house (yard, roof, garden)?

Yes

No

18. Did you do any physical activity inside the house (cleaning the house, preparing food, ironing, and washing clothes) before the pandemic?

Yes

No

19. Did you do any kind of sport (walking, resistance training, biking, swimming...) before the pandemic?

No (move to Question No. 23)

Rarely (move to Question No. 21)

Sometimes (move to Question No. 21)

Regularly (move to Question No. 20)

20. How many times per week?

Once

Two to three times

Four to five times

Daily

21. What kind of sport did you do before the pandemic? (you can choose more than one answer)

Walking

Bike riding

Resistance exercises

Dancing

Playing football

Swimming

Other (mention it)

22. Where did you play sports before the pandemic?

Inside the house

Outside the house

23. Do you do any physical activity inside the house (cleaning the house, preparing food, ironing, and washing clothes) during the pandemic period?

Yes

No

24. Did you do any kind of sport (walking, resistance training, biking, swimming ...) during the pandemic period?

No (move to Question No. 28)

Rarely (move to Question No. 26)

Sometimes (move to Question No. 26)

Regularly (move to Question No. 25)

25. How many times per week?

Once

Two to three times

Four to five times

Daily

26. What kind of sport did you do during the pandemic? (you can choose more than one answer)

Walking

Bike riding

Resistance exercises

Dancing

Playing football

Swimming

Other (mention it)

27. Where did you play sports during the pandemic?

Inside the house

Outside the house

28. Your sleep hours during the day during the curfew period:

Less than 4 hours

5-6 hours

7-9 hours

More than 9 hours

29. Did your eating habits change during the curfew period?

Yes (move to Question No. 30)

No (move to Question No. 31)

30. How did your eating habits change? (you can choose more than one answer)

Increase your daily food intake

Reducing your daily food intake

Increase your consumption of frozen and canned foods

Reducing the consumption of frozen and canned foods

Increase the consumption of vegetables and fruits

Reducing the consumption of vegetables and fruits

Preparing baked goods (pastries) and sweets at home

Eating more fast food

Reducing fast food intake

Use of nutritional supplements and vitamins

Other (mention it)

31. Did you notice any change in weight during the pandemic period?

Increase (move to Question No. 32)

Decrease (move to Question No. 34)

No change (move to the end of the questionnaire)

32. The amount of the increase in kilograms:

From 1 to 2 kg

From 3 to 4 kg

From 5 to 6 kg

More than 6 kg

I do not know

33. In your opinion, what are the reasons that led to your weight gain? (you can choose more than one answer)

Psychological state (fear, anxiety, psychological stress)

Feeling bored and empty

Sleep disturbances

Not feeling full

Eating more snacks

Eat fast food

Lack of movement and increased sitting hours on TV or mobile phones

Close sports clubs

Lack of availability of fresh vegetables and fruits

Chronic diseases

Other (mention it)

34. The amount of decrease in kilograms:

From 1 to 2 kg

From 3 to 4 kg

From 5 to 6 kg

More than 6 kg

I do not know

35. In your opinion, what are the reasons that led to your weight loss? (you can choose more than one answer)

Psychological state (fear, anxiety, psychological stress)

Long or late working hours

Sleep disturbances

Reducing the number of meals per day

Cut out fast food

Commitment to healthy food

Maintain home exercises

chronic diseases

Other (mention it)

End of questionnaire
